# Vacuolar Protein-Sorting Receptor MoVps13 Regulates Conidiation and Pathogenicity in Rice Blast Fungus *Magnaporthe oryzae*

**DOI:** 10.3390/jof7121084

**Published:** 2021-12-17

**Authors:** Xueming Zhu, Lin Li, Jiaoyu Wang, Lili Zhao, Huanbin Shi, Jiandong Bao, Zhenzhu Su, Xiaohong Liu, Fucheng Lin

**Affiliations:** 1 State Key Laboratory for Managing Biotic and Chemical Threats to the Quality and Safety of Agro-Products, Institute of Plant Protection and Microbiology, Zhejiang Academy of Agricultural Sciences, Hangzhou 310021, China; zhuxm@zaas.ac.cn (X.Z.); 11816015@zju.edu.cn (L.L.); wangjiaoyu78@sina.com (J.W.); lilizhao2089@163.com (L.Z.); baojiandong@gmail.com (J.B.); 2 State Key Laboratory of Rice Biology, China National Rice Research Institute, Hangzhou 311499, China; shihuanbin@caas.cn; 3 State Key Laboratory of Rice Biology, Institute of Biotechnology, Zhejiang University, Hangzhou 310058, China; zzsu@zju.edu.cn (Z.S.); xhliu@zju.edu.cn (X.L.)

**Keywords:** *Magnaporthe* *oryzae*, MoVps13, ER-phagy, pathogenic fungi, cell wall integrity

## Abstract

*Magnaporthe oryzae* (synonym *Pyricularia oryzae*) is a filamentous fungal pathogen that causes major yield losses in cultivated rice worldwide. However, the mechanisms of infection of *M. oryzae* are not well characterized. The VPS13 proteins play vital roles in various biological processes in many eukaryotic organisms, including in the organization of actin cytoskeleton, vesicle trafficking, mitochondrial fusion, and phagocytosis. Nevertheless, the function of the Vps13 protein in plant pathogenic fungi has not been explored. Here, we analysed the biological functions of the Vps13 protein in the development and pathogenicity of *M.* *oryzae*. Deletion mutants of MoVps13 significantly reduced the conidiation and decreased the rate of fungal infection on hosts. Moreover, the loss of MoVps13 resulted in defective cell wall integrity (CWI) and plasma membrane (PM) homeostasis when treated with chemicals for inducing cell wall stress (200 mg/mL Congo Red or 0.005% SDS) and sphingolipid synthesis inhibitors (2 μM myriocin or 2 μM amphotericin B). This indicated that MoVps13 is also involved in cell wall synthesis and sphingolipid synthesis. Through immunoblotting, autophagic flux detection, co-localization, and chemical drug sensitivity assays, we confirmed the involvement of Movps13 in ER-phagy and the response to ER stress. Additionally, we generated the C-terminal structure of MoVps13 with high accuracy using the alphaflod2 database. Our experimental evidence indicates that MoVps13 is an important virulence factor that regulates the pathogenicity of *M.* *oryzae* by controlling CWI, lipid metabolism and the ER-phagy pathway. These results have expanded our knowledge about pathogenic fungi and will help exploration for novel therapeutic strategies against the rice blast fungus.

## 1. Introduction

The filamentous fungus *Magnaporthe oryzae* (syn *Pyricularia oryzae)* causes rice blast disease, which is responsible for a significant reduction in rice production and poses a serious threat to rice global food security [1,2,3]. *Magnaporthe oryzae* exhibits classic characteristics in terms of growth, development, and plant infection. The typical disease cycle starts with three celled conidia [4,5]. The conidia attach to the surface of host leaves by powerful glycoprotein-rich mucilage and germinate within a few hours to form germ tubes [6,7]. At the tip of the germ tube, melanized and pressurized dome-shaped infected cells, called appressoria, are formed [8,9]. Intracellular nutrients, such as glycogen and glycerol, are continuously decomposed by autophagy and transported to the appressorium, which develops enormous turgor that exerts a physical force to break the waxy cuticle of the rice leaf and subsequently differentiates into biotrophic invasive hyphae (IH) [10,11,12,13]. Primary IH develop into bulbous pseudohyphae in rice cells and then expand to other cells by filamentous hyphae [14,15]. Understanding the mechanism of infection for rice blast fungus may help inform strategies to control rice blast disease.

VPS13 proteins are large, evolutionarily conserved proteins found in many eukaryotes, which play vital roles in organization of actin cytoskeleton, vesicle trafficking, phagocytosis, and autophagy [16,17,18]. In humans, there are four VPS13 proteins (HsVps13A, HsVps13B, HsVps13C and HsVps13D) [19,20,21,22]. These proteins are partly redundant but have independent functions. Deletion of HsVPS13A was shown to lead to an autosomal recessive disorder characterized by adult-onset chorea, progressive neurodegeneration, and abnormal erythrocyte morphology (acanthocytosis) [23]. Loss of HsVps13B caused a rare autosomal recessive disorder characterized by non-progressive psychomotor retardation, microcephaly, characteristic facial features, retinal dystrophy, and intermittent neutropenia in children [24,25,26]. Genetic studies have also revealed that variants of HsVps13C are associated with increased risk of diabetes, and HsVps13D contributes to septic shock mortality [27,28]. There is only a single Vps13 protein in *Saccharomyces cerevisiae*. In yeast, Vps13 is mainly localized in the endoplasmic reticulum (ER)-mitochondria contact site and the ER-nuclear vacuole junction. However, the functions of Vps13 and the regulatory mechanism of its localization are not clear [16,29]. Recent studies have found that Vps13 is required for the packaging of ER into autophagosomes during ER-phagy in yeast, indicating that Vps13 may participate in the degradation pathway [17,30]. As for yeast, only a single Vps13 protein was found in the plant pathogenic fungus *M. oryzae*. Nevertheless, the biological role of MoVps13 is not well characterized. In this study, we identified the Vps13 protein and analysed its biological functions in hyphae growth, conidia production, appressorium formation, and plant infection by *M. oryzae*.

Most chemical drugs are designed based on the three-dimensional structure of target proteins. However, determining the protein structure using experimental methods is a challenge for a vast majority of proteins, despite steady advances in x-ray crystallography, nuclear magnetic resonance (NMR), and cryo-electron microscopy (cryo-EM) [31,32,33]. Despite the extensive efforts of the global scientific community over recent decades, experimentally determined structures are available for only ~18% of the total residues in protein sequences in the human proteome [32]. In other organisms, especially plant pathogenic fungi, only a few protein structures have been analysed, which is a key barrier to the development of new fungicides. The ability to characterize protein structure has substantially improved with the use of structural modelling using the machine-learning algorithm, AlphaFold2, an artificial intelligence system developed by DeepMind, that predicts the 3D structure of a protein from its amino acid sequence. Its predictive accuracy has been shown to be comparable to that of experiments [31,32,34]. In our research, we constructed the SHR, Vps13_C and ATG_C domain 3D structure of MoVps13 with high confidence using AlphaFold2 software. This result will help better characterize the function of MoVps13 in rice blast fungus and may serve as an experimental platform for drug testing based on protein structure. Future research will entail screening of small molecule compounds based on the 3D structure of MoVps13.

## 2. Materials and Methods

### 2.1. Gene Deletion and Complement Strategy

For the gene knockout, we used homologous recombination strategies modified by Lu et al. [35]. The knockout vector PKO3A, containing a suicide gene, *HSVtk*, was cut by the restriction enzymes *Xba*I and *Hid*III (New England Biolabs, Beijing, China). The ~1.2 kb upstream fragment and the fragment downstream of the target gene were amplified with specific primers (Table S1), and then fused with the resistant gene *HPH* and linearized by recombinase Exnase (Vazyme Biotech Co., Ltd., C113-02, Nanjing, China). The recombination plasmids were transferred into *Agrobacterium tumefaciens* and the knockout was performed by *At*MT (*Agrobacterium tumefaciens* mediated transformation) methods. The mutants were screened using a CM medium containing 200 μg/mL hygromycin B and 0.5 μM 5-fluoro-2′-deoxyuridine and further verified by multiple rounds of PCR and RT-PCR, as described by Zhu et al. [11].

For the mutant recovery assay, the full-length target gene was fused to the PKD5-GFP vector, which contains a sulfonylurea resistance gene (*SUR*), and transformed into the mutant. The complementation strains were identified by phenotype recovery condition, fluorescence observation, and quantification of mRNA expression.

### 2.2. Strains, Growth Conditions, and Phenotypic Analyses

All strains of *M. oryzae* used in this work were Guy11 and cultured on a complete medium under 16 h/8 h light/dark cycle at 25 °C [36]. For the virulence assay, the mycelium plugs were incubated on cut leaves of rice and barley for 4 days at 25 °C in more than 95% humidity conditions. For spore incubation assay, the conidial suspensions (5 × 10^4^ conidia/mL) were sprayed on 14 day old rice seedlings (CO39) and observed at 7 days post-incubation. The pathogenic lesions were quantified using ImageJ software.

### 2.3. Growth Stress Assay

To test the response of mutants to different stresses, different inhibitors were added to a solid CM medium with appropriate quantification. All assays in this study were performed in triplicate. The relative growth rates were calculated using the formula: growth rate = (the diameter of the strain treated with chemicals)/(the diameter of the untreated strain).

### 2.4. Autophagy Assays

To detect autophagy flux, the GFP-MoAtg8 were transformed to the wild type Guy11 and the Δ*Movps13* mutant using an in situ complementary method. The Guy11::GFP-MoAtg8 and the Δ*Movsp13*::GFP-MoAtg8 strain were grown in liquid CM medium for 48 h with 150 rpm shaking at 25 °C, and shifted to a SD-N medium for induction for 4 h. For the ER-phagy assay, the MoSec63-GFP were transformed to the wild type Guy11 and the Δ*Movps13* mutant, and the Guy11::MoSec63-GFP and the Δ*Movsp13*::MoSec63-GFP strains were grown in liquid CM medium for 48 h with 150 rpm shaking at 25 °C, then shifted to a SD-N medium for induction for 4 h with 5 mM DTT, as described by Wei et al. [37].

### 2.5. Immunoblotting Analysis

For macroautophagy and ER-phagy, the free GFP and fusion bands were detected by GFP antibody (GFP 1:10,000; Abcam; ab32146, Shanghai, China) with 12% SDS-PAGE. For the MoAtg8 and the MoAtg8-PE turnover assays, the MoAtg8 and the MoAtg8-PE bands were detected using an Atg8 antibody (1:2000, BML; PM090, Beijing, China) with 13.5% SDS-PAGE. For detecting the phosphorylation level, proteins of the Guy11 and Δ*Movps13* mutant strains were extracted by TCA-SDS methods. The Mps1 phosphorylation level was detected using a MAPK antibody (Cell Signaling Technology; 9212S, Danvers, MA, USA).

## 3. Results

### 3.1. Identification of VPS13 Protein in M. oryzae

**Figure 1:**
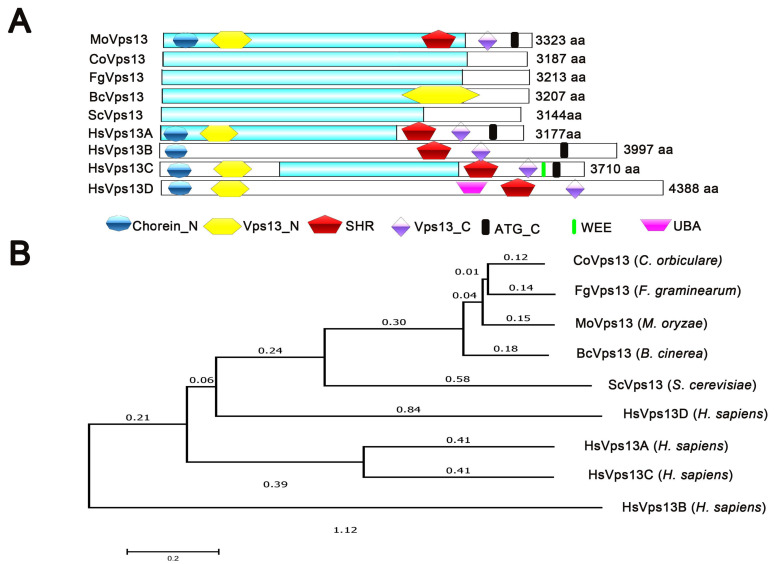
Characterization of Vps13 protein in *M. oryzae.* (**A**) Schematic illustration of the Vps13 proteins in *M. oryzae*, *C. orbiculare*, *F. graminearum*, *B. cinerea*, *S. cerevisiae,* and *H. sapiens*. (**B**) Phylogenetic tree of the Vps13 proteins in different organisms. The phylogenetic tree was constructed using the neighbour-joining method with 1000 bootstrap replicates in MEGA software.

In *M*. *oryzae*, a single Vps13 domain-containing protein, MGG_06537, was identified in the genome by the EnsemblFungi database (http://fungi.ensembl.org/Magnaporthe_oryzae, accessed on 5 July 2020). We assigned the MoVps13 to *M*. *oryzae*. Pfam domain analysis showed that MoVps13 has 3223 amino acids, containing Chorein_N, Vps13_N domain in the N-terminal and SHR, Vps13_C and the ATG_C domain in the C-terminal. All domains are conserved in humans, *S*. *cerevisiae* and other plant pathogenic fungi, such as *Fusarium graminearum*, *Colletotrichum orbiculare,* and *Botrytis cinerea* (Figure 1A). Phylogenetic analysis showed a high similarity of MoVps13 to ascomycetes fungi, such as *C. orbiculare* CoVps13, *F. graminearum* FgVps13, and *B. cinerea* BcVps13. We also found that MoVps13 is similar to *S. cerevisiae* ScVps13, *H. sapiens* HsVps13D, HsVps13A, HsVps13C, and HsVps13D (Figure 1B). These results suggested that Vps13 is conserved in many eukaryotes.

### 3.2. Three-Dimensional Structure of MoVps13 Protein in M. oryzae

**Figure 2:**
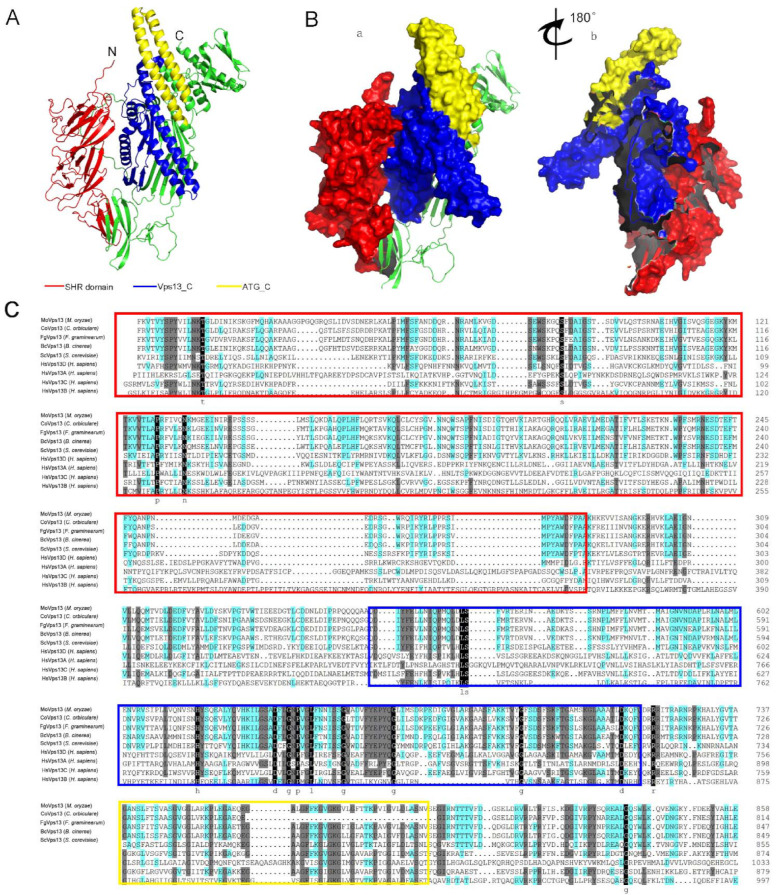
Three-dimensional structure of the MoVps13 protein in *M. oryzae* (**A**) The MoVps13 C-terminal 2279-3223 amino acid sequence generated by the structure prediction server AlphaFold2 database (https://www.cloudam.cn/v2/console/create-job, accessed on 15 October 2021). The illustration of the MoVps13 C-terminal was constructed using PyMol software. N, N-terminus; C, C-terminus. Red, SHR domain; Blue, Vps13_C domain; Yellow, ATG_C domain. (**B**) a, Surface diagram of the MoVps13 C-terminal model. Red indicates the SHR domain surface, blue indicates the SHR domain surface, and yellow indicates the SHR domain surface. b, The right panel shows the diagram that was rotated 180° about the indicated axis. (**C**) Alignment of the Vps13 MoVps13 C-terminal using Clustal X.

For designing drugs to control pathogenic fungi and better understand the protein function, structural data at atomic resolution are required. However, structural information for most proteins is still limited in the Protein Data Bank. AlphaFold2 is a recently developed protein predictive platform based on the machine learning method, which has greatly expanded the structural coverage of sequences, with high accuracy. To obtain the structure of MoVps13, we submitted the MoVps13 C-terminal 2279-223 amino acid sequence to the structure prediction server AlphaFold2 database (https://www.cloudam.cn/v2/console/create-job, accessed on 15 October 2021). The mean pLDDT values, up to 78.18, indicated that the predicted structure was modelled well. In our predicted model, the SHR domain contains 19 β-sheets, the MoVps13_C domain contains 4 α-helices, and the ATG_C domain has only two α-helices (Figure 2A,B). Next, we analysed the Vps13 C-terminal 2279-3223 amino acid sequences using Clustal X software with the multiple sequence alignment method. The results showed that the SHR domain, vps13_C, and the ATG_C domain are conserved in different organisms (Figure 2C).

### 3.3. MoVps13 Is Involved in Conidiation and Virulence

**Figure 3:**
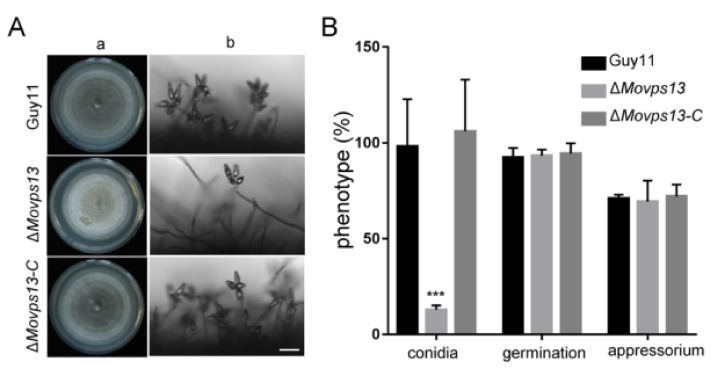
MoVps13 is involved in conidiation. (**A**) a, Observation of growth conditions of the Guy11, the Δ*Movps13* mutant, and the complementation strains in a CM medium at 25 °C for 7 days. b, Microscopic morphology of the Guy11, the Δ*Movps13* mutant, and the complementation strains. Bar = 50 μm. (**B**) Conidiation, conidium germination, and appressorium formation statistics in the Guy11, the Δ*Movps13* mutant, and the complementation strains; data analysed using prism 7.0 software. Asterisks indicate statistically significant differences (*t* test, *** *p* < 0.01).

**Figure 4:**
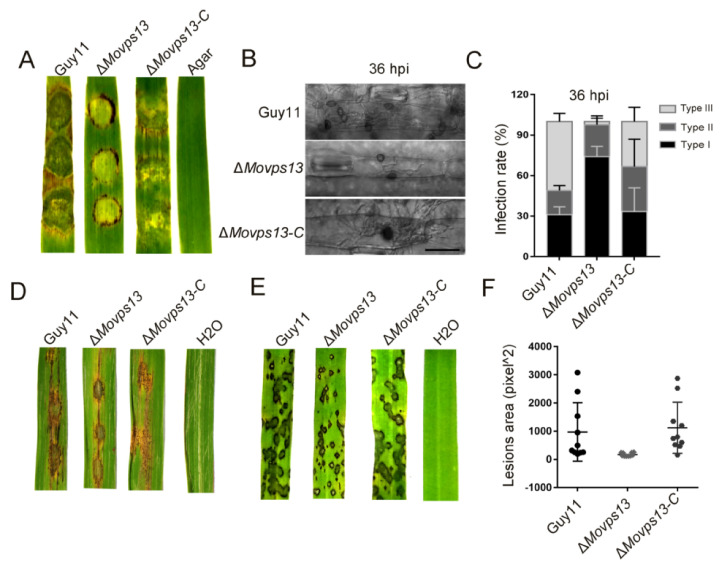
Virulence analyses for the Δ*Movps13* mutant. (**A**) Pathogenic lesions on cut leaves of barley with mycelial plugs from the Guy11, the Δ*Movps13* mutant, and the complementation strains. (**B**) Penetration assays on barley leaves within the Guy11, the Δ*Movps13* mutant, and the complementation strains. Bar = 50 μm. (**C**) Three different types of invasive hyphae were quantified and statistically analysed in the Guy11, the Δ*Movps13* mutant*,* and the complementation strains. Error bars represent the SD. Type I, infection hyphae were restricted to the first invaded host, Type II, no infection hyphae, Type III, infection hyphae were expanded to neighbouring cells (**D**) Disease lesions on wound leaves of rice in the Guy11, the Δ*Movps13* mutant, and the complementation strains. (**E**) Rice spraying assays to detect pathogenicity. (**F**) Statistical analysis of the lesions area of the Guy11, the Δ*Movps13* mutant, and the complementation strains using ImageJ software.

Previous studies have found that the Vps13 protein plays vital roles in diverse organisms ranging from yeasts to humans. Given it is a conserved protein, we assumed that MoVps13 also has biological roles in *M. oryzae*. To explore the functions of MoVps13 in *M. oryzae*, we obtained knockout mutants of *MoVPS13* by a high-throughput target-gene deletion method (Figure S1A,B). After confirming the mutants by PCR and the qPCR method as described by Lu et al., we subsequently complemented the mutants with the *MoVPS13* gene [38]. The vegetative growth of the *MoVPS13* deleted mutant was similar to the wild type; however, the conidiation ability of the Δ*Movps13* mutant was significantly lower (Figure 3A,B). Next, we tested whether the germination and appressorium formation were affected in the Δ*Movps13* mutant. The conidium germination and appressorium formation were normal in the Δ*Movps13* mutant after 6, 12, 24 h induced in artificial hydrophobic film (Figure 3B). To examine whether MoVps13 regulates the pathogenicity of *M. oryzae*, virulence assays were performed on two different susceptible plant hosts (rice and barley). Mycelial plugs of the Guy11, the Δ*Movps13*, and the complementation strains were inoculated on detached barley leaves. At 4 day-post-incubation (dpi), the wild type and the complementation strains caused severe lesions, while only small lesions could be detected in the Δ*Movps13* mutant (Figure 4A). Simultaneously, an infection assay was performed to gain further insights into the effects of the Δ*Movps13* mutant on disease progression. After 36 h of inoculation, almost all infection hyphae in the wild type Guy11 and the complemented strains showed numerous branches that had expanded to neighbouring cells. However, only a few infection hyphae in the Δ*Movps13* mutant expanded to other cells and were restricted to the first invaded host (Figure 4B,C). To test the conidia pathogenicity of the Δ*Movps13* mutant, we inoculated conidial suspensions (5 × 10^4^ conidia/mL) of wild-type, the Δ*Movps13*, and the complementation strain to wound-treated rice leaves and sprayed the conidial suspensions (5 × 10^4^ conidia/mL) to 14-day rice seedlings (CO39). After 7 dpi, the Guy11 and complementation strain caused large expansive lesions on rice leaves, but the Δ*Movps13* mutant only produced restricted lesions (Figure 4D–F). These results show that MoVps13 has a role in conidiophore formation, conidial differentiation, invasive hyphal growth, and host colonization.

### 3.4. MoVps13 Localized in ER and Involved in ER-Phagy

**Figure 5:**
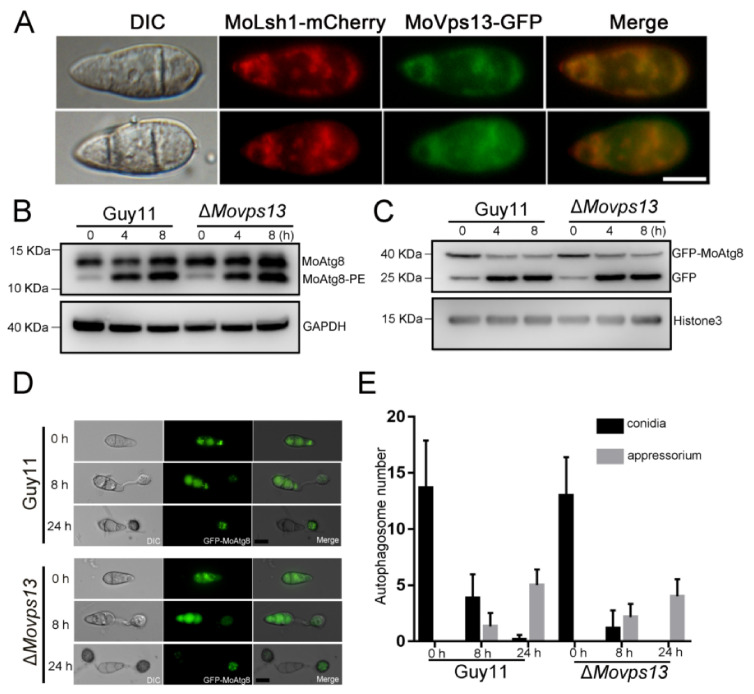
Autophagy flux analysis in Δ*Movps13*. (**A**) Co-localization of MoVps13 with MoLsh1 in conidia. The fluorescence was observed by fluorescence microscopy. Bar = 10 μm. (**B**) Immunoblot analysis of MoAtg8/MoAtg8-PE turnover in the Guy11 strain and the Δ*Movps13* mutant. (**C**) Immunoblot analysis of GFP-MoAtg8 proteolysis in the Guy11 strain and the Δ*Movps13* mutant. (**D**) Observation of autophagosomes in conidia in the Guy11 strain and the Δ*Movps13* mutant at 0 h, 8 h, and 24 h. Bar = 10 μm. (**E**) Statistics were calculated using ImageJ software and Prism software.

To further explore the functions of MoVps13 in *M. oryzae*, the *MoVPS13-GFP* was constructed and transformed into the Δ*Movps13* mutant. Under fluorescence microscopy, MoVps13 appeared in an ER-like structure of hyphae and conidia. To confirm the natural structures of MoVps13, MoLhs1-mCherry (an ER marker) fusion protein was co-expressed with MoVps13-GFP. The localization of MoVps13 coincided completely with MoLhs1-mCherry in the hyphae and conidia, indicating that MoVps13 was mainly localized in the ER (Figure 5A). In *Dictyostelium discoideum* and human HeLa cells, VPS13A regulates autophagy; VPS13A downregulation has been shown to cause GFP-LC3 and GFP-WIPI1 accumulation [39]. To examine whether MoVps13 regulates macroautophagy, the autophagy flux was detected in wild type Guy11 and the Δ*Movsp13* strain. In nutrition condition, autophagy was weak, and few MoAtg8-PE (autophagy marker to monitor autophagy flux) band were found. Deprivation of the nitrogen source induced autophagy resulted in MoAtg8-PE accumulation (Figure 5B). Next, we tested the activity of GFP-MoAtg8 in nutrition and nitrogen starvation conditions. Both the Guy11 and the Δ*Movps13* strains increased free GFP bands when induced in a SD-N medium for 4 h; however, there was no significant difference between the Guy11 and the Δ*Movps13* strains (Figure 5C). Autophagy plays a key role in pressure accumulation during formation of the appressorium. To verify the role of MoVps13 in infection-associated autophagy in *M. oryzae*, we observed the numbers of autophagosomes in conidia of the wild-type Guy11 and the Δ*Movps13* strain in nutrition starvation conditions for 0, 8 and 24 h. The GFP-MoAtg8 puncta appeared when induced for 0 h, and most of the free GFP fluorescence in conidia occurred in vacuoles at 8 h and 24 h. At 24 h, no GFP fluorescence was found and most of GFP-MoAtg8 puncta were in the appressorium, both in the wild-type Guy11 and the Δ*Movps13* strain. However, there was no significant difference between the Guy11 and the Δ*Movsp13* strain with respect to conidia, or the appressorium (Figure 5D,E).

**Figure 6:**
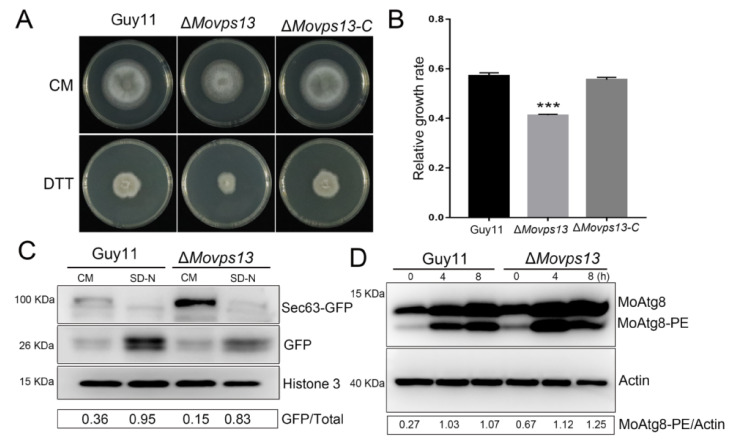
Involvement of MoVps13 in ER-phagy process. (**A**) Growth of the Guy11, the Δ*Movps13* mutant, and the complementation strains on a CM agar medium containing 5 mM DTT for 5 days at 25 °C. (**B**) The relative growth rates of the Guy11, the Δ*Movps13* mutant*,* and the complementation strains. Asterisks indicate statistically significant differences (*** *p* < 0.01). (**C**) Immunoblot analysis of MoSec63-GFP proteolysis in the Guy11 strain and the Δ*Movps13* mutant. The degradation rates were calculated by the formula: GFP/(GFP+MoSec63-GFP) (**D**) Immunoblot analysis of MoAtg8/MoAtg8-PE turnover in the Guy11 strain and the Δ*Movps13* mutant after treatment with 5 mM DTT.

Recent studies have shown that Vps13 is required for the packaging of the ER into autophagosomes during ER-phagy in *S. cerevisiae* [30]. We further detected whether ER-phagy was affected in the Δ*Movps13* strain. When treated with 5 mM DTT (a chemical inducer of ER-phagy) on the CM medium, the Δ*Movps13* strain showed greater sensitivity than the wild-type and the complementation strain for 5 days (Figure 6A,B). Next, we transferred Sec63-GFP (the ER-Phagy marker) into the Guy11 and the Δ*Movps13* strains and induced the transformants with 5 mM DTT in a liquid CM medium for 4 h. In the CM medium, the sec63-GFP was found and few free GFP bands could be detected in both the wild type and the Δ*Movps13* strains; however, the Sec63-GFP bands were stronger in the mutant than in the Guy11 strain. After induction for 4 h, a few Sec63-GFP were found and the free GFP band was increased in the Guy11 strain. However, the Sec63-GFP band still persisted, while the free GFP was lower in the Δ*Movps13* strain compared to the wild type (Figure 6C). Although the MoAtg8 and the MoAtg8-PE were increased when induced by DTT, there was no significant difference between the Guy11 and the Δ*Movps13* strains (Figure 6D). These results suggested that Δ*Movsp13* is mainly involved in ER-phagy rather than macroautophagy.

### 3.5. MoVps13 Is Hypersensitive to Sphingolipid Synthesis Inhibitors and Displays Cell Wall Defects

**Figure 7:**
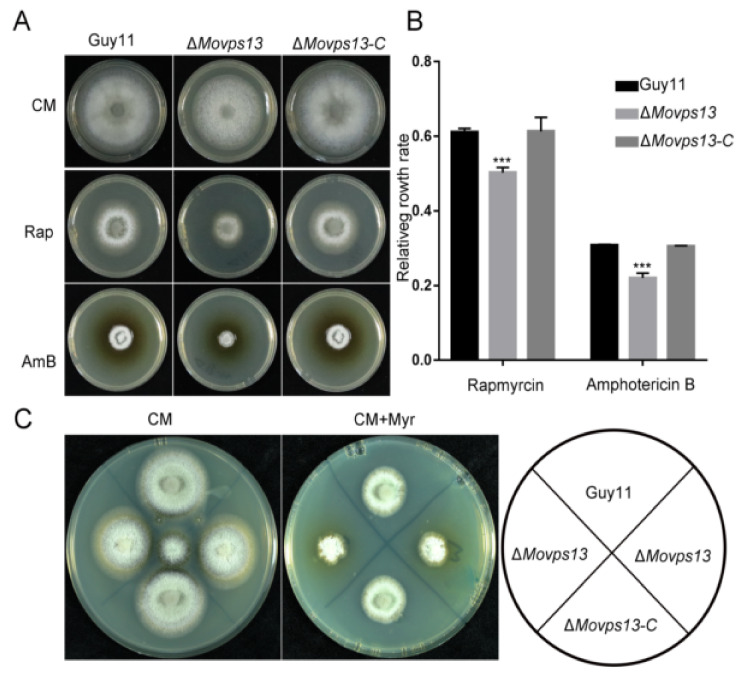
Lipid homeostasis was disturbed in the Δ*Movps13* mutant. (**A**) Growth of the Guy11, the Δ*Movps13* mutant, and the complement strains on a CM agar medium containing 100 ng/mL rapamycin or 2 μM amphotericin B for 5 days at 25 °C. (**B**) Relative growth rates of the Guy11, the Δ*Movps13,* and the complement strains. Asterisks indicate statistically significant differences (*** *p* < 0.01). (**C**) Growth of the Guy11, the Δ*Movps13* mutant, and the complement strains on CM agar medium containing 2 μM myriocin for 5 days at 25 °C.

**Figure 8:**
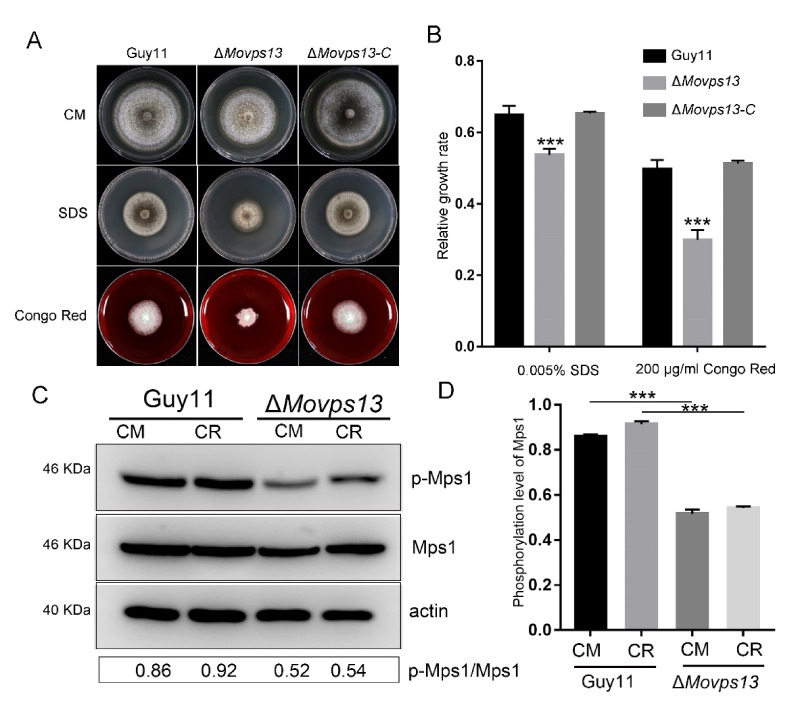
Involvement of MoVps13 in the CWI pathway. (**A**) Growth of the Guy11, the Δ*Movps13* mutant, and the complement strains on a CM agar medium containing 200 μg/mL Congo Red or 0.005% SDS for 5 days at 25 °C. (**B**) Relative growth rates of the Guy11, the Δ*Movps13* and the complement strains. Asterisks indicate statistically significant differences (*** *p* < 0.01). (**C**) Western blot analysis of the phosphorylation of MoMps1 in the Guy11 and the Δ*Movps13* mutant strains. (**D**) The phosphorylation level of MoMps1 was analysed using ImageJ software. Asterisks indicate statistically significant differences (*** *p* < 0.01).

Previous studies have shown that Vps13 is a lipid transfer protein involved in non-vesicular traffic of secretory lipids from the ER in *S*. *cerevisiae* [40]. To verify the functions of MoVps13 in lipid synthesis, we examined the growth of the Δ*Movps13* mutant in the presence of a TOR kinase inhibitor (rapamycin), sterol lipid binding inhibitor (amphotericin B), and a sphingolipid synthesis inhibitor (myriocin). The Δ*Movps13* mutant showed greater sensitivity to all three inhibitors compared with the wild-type Guy11 and the complementation strains, indicating that loss of Vps13 impaired the PM homeostasis in *M. oryzae* (Figure 7A–C). Next, we further examined whether MoVps13 participates in the cell wall synthesis pathway by performing cell wall stress assays in the wild-type Guy11, the Δ*Movps13* mutant, and the complementation strains. When treated with 0.005% SDS and 200 μg/mL Congo Red in CM medium for 7 days, the growth rates of the Δ*Movps13* mutant were significantly reduced, both by SDS and Congo Red compared with the wild-type Guy11 and the complementation strains (Figure 8A,B). To verify the regulation of the CWI pathway, the phosphorylation level of MoMps1 was detected by Western blot, which showed that the phosphorylation level of Mps1 was significantly decreased in the Δ*Movps13* mutant irrespective of CM conditions or the Congo Red stress conditions (Figure 8C,D). These results suggested that MoVps13 has key roles in lipid synthesis and is involved in the cell wall synthesis pathway.

## 4. Discussion

*Magnaporthe oryzae* has the ability to cause rice blast disease via a penetration peg that is produced by the appressorium and its expansive growth across cells [10,41]. These invasion processes are regulated by a variety of signal pathways and effectors between plants and pathogens. In recent years, Vps13 proteins have been reported to play vital roles in regulating actin cytoskeleton, vesicle trafficking, phagocytosis, and autophagy from yeast to humans [18,21,30,42]. However, there is little research on the functions of Vps13 proteins in filamentous fungi. Whether Vps13 plays a role in the growth, development, and virulence of *M. oryzae*, is not clear. To explore these scientific issues, we deleted the *MoVPS13* gene in wild type strain Guy11 and systematically analysed its effect on the growth, development, and pathogenicity of *M. oryzae*. In this work, we characterized MoVps13 in *M. oryzae* and showed that it is required for fungal development and plant pathogenicity. We discovered that VPS13 is involved in ER-phagy and is mainly localized in the ER, which is consistent with that observed in other organisms. Additionally, we also found that MoVps13 participates in maintaining cell wall integrity, lipid homeostasis, and regulates TOR activity. To the best of our knowledge, these results have not been reported for other species, and these discoveries will help us better understand the various functions of Vps13 protein in plant pathogenic fungi.

Macroautophagy (referred to as ‘autophagy’ hereafter for simplicity) is an evolutionarily conserved cellular pathway in many eukaryotic organisms. In this process, some of the damaged proteins or organelles are swallowed by autophagic vesicles and transferred to lysosomes (animals) or vacuoles (fungi and plants) for degradation and recycling [43,44]. In recent decades, autophagy has been shown to be an important factor that regulates host infection by many plant pathogenic fungi [11,43,44,45,46,47]. In *M. oryzae*, autophagy was shown to promote appressoria formation and pressure accumulation. Knockout of the core autophagy genes was shown to lead to complete loss of pathogenicity [10,44]. In *F. graminearum*, autophagy is required for proper vegetative growth, full virulence, and toxin biosynthesis [48]. In *U. maydis*, autophagy pathways are important for normal budding of haploid sporidia, survival under nutrient starvation, and pathogenic development [47]. Recent studies have indicated an essential role of selective autophagy, such as mitophagy, ER-phagy and lipophagy, in cellular development [49,50]. Sandra et al. first found that a Vps13A homologous protein TipC is required for autophagic flux in *Dictyostelium discoideum*. Lack of TipC resulted in a reduced number of autophagosomes and impaired autophagic degradation as determined by a proteolytic cleavage assay [39]. Nevertheless, no autophagy defects have been reported in the mutant strain so far, despite there being only one Vps13 protein in yeast [51]. Recently, Chen et al. reported that the Δ*vps13* mutant is defective in the selective autophagy of ER (ER-phagy), an alternate ER degradation pathway that engulfs ER into autophagosomes and delivers them to lysosomes or vacuoles for degradation [30]. These results indicated that the Vps13 family protein may have unique functions in different organisms. *M. oryzae* has only one Vps13 protein. However, whether MoVps13 participates in the autophagy pathway is still unclear. To answer this question, we detected the autophagy flux using GFP-Atg8 and Atg8/Atg8-PE turnover assays. In our study, we found that the number of autophagosomes and autophagic degradation was not significantly changed compared with the wild type, which indicated the lack of involvement of MoVps13 in macroautophagy. The GFP-Movps13 showed that MoVps13 is localized in the ER. Therefore, we also tested the ER-phagy conditions in Δ*Movps13* using the ER-phagy marker Sec63-GFP that is described by Sun et al. [52]. In the Δ*Movsp13* mutant, the band Sec63-GFP was maintained at a high level and fewer free GFP bands were found compared with the wild type Guy11 strain after treatment with DTT to induce ER stress; these findings indicated that MoVps13 promoted ER-phagy in *M*. *oryzae*.

We also observed a significant decrease in the mycelial growth of the Δ*Movps13* mutant in the presence of SDS, Congo Red, and Rapamycin. Loss of MoVps13 led to production of few aerial hyphae and the middle sections of old hyphae collapsed when cultured on a CM medium for 10 days (data not shown). This phenomenon was similar to that observed in the Mps1 deletion mutant, suggesting impairment of the cell wall integrity (CWI) in the Δ*Movps13* mutant. The fungal cell wall has a complex structure and plays a key role in modulating the response of fungal pathogens to host and environmental signals, as well as in plant infection [53,54,55,56]. In *M*. *oryzae*, the cell wall synthesis is mainly regulated by the MAPK-Mps1 pathway, which is important for cell wall integrity, conidiogenesis, and plant infection [54,55]. The MoMps1 deletion mutant was shown to exhibit defective appressorium penetration and autolysis defects with fewer aerial hyphae, rare conidiation, and loss of virulence [57,58]. A recent study demonstrated the crosstalk between the CWI pathway and TOR signalling, which has recently been recognized as an important regulator of rice infection by the blast fungus *M. oryzae* [59]. The TOR interaction protein Tap42 interacts with MoTip41 and mediates the crosstalk between the TOR and the CWI signalling pathways [60]. Our results demonstrate that MoVps13 affects both the CWI pathway and TOR signalling and may regulate cell wall integrity by promoting TOR activity. The precise nature of the relationships needs to be confirmed by further research.

Our previous study found that TOR signalling mediates MoYpk1 activity, which is a response to PM homeostasis [43]. The PM is a selectively permeable barrier and a dynamic interface that helps maintain cellular environmental homeostasis [43,61]. Sterols and sphingolipids, as the main components of the cell membrane, play a very important role in maintaining cell membrane homeostasis and intracellular signal transmission, and are also ideal targets for novel fungicides [62,63,64,65]. Recent studies have shown that membrane lipids are synthesized in the ER by multiple biosynthetic enzymes, and then transferred to other intracellular membrane systems via vesicular or non-vesicular trafficking pathways [40,43]. In yeast, sterols and sphingolipids are synthesized in the ER by a series of catalysing enzymes and delivered to the PM or other membrane organelles [66]. Vps13 is a lipid transfer protein that is involved in non-vesicular traffic of secretory lipids from the ER [40]. Given the regulatory effect of MoVps13 on TOR activity and its localization in the ER, we speculate that MoVps13 is also involved in maintaining PM homeostasis in *M. oryzae*. To our surprise, the Δ*Movps13* mutant showed increased sensitivity to myriocin (a sphingolipid synthesis inhibitor) and amphotericin B (which binds to PM sterols and disrupts PM structure), indicating the involvement of MoVps13 in PM homeostasis.

Research on the pathogenicity mechanism of *M. oryzae* contributes to the development of novel strategies to control rice blast, such as improving cultivation methods, breeding disease-resistant varieties, and designing new antifungal drugs [67]. Until now, the use of effective antifungal drugs is a key component of management of fungal pathogenic disease. However, there is a paucity of antifungal agents that can help prevent these diseases [68]. Unfortunately, the development of fungicide resistance outpaces the discovery of new fungicides. Therefore, identification of new targets for disease management is a key imperative. Recently, He et al. found several anti-penetrant drugs (metazachlor, cafenstrole, and diallate), acted as very-long-chain fatty acids VLCFA biosynthesis inhibitors, which showed effective, broad-spectrum fungicidal activity against diverse fungal pathogens, without affecting their respective hosts. Their results provided a class of fungicides which offer broad prospects for control of plant diseases [67]. With the advances in artificial intelligence technology, designing and screening of drugs based on protein structure for control of fungal diseases is an emerging research hotspot. In our study, we identified the SHR, Vps13_C and ATG_C domain structures of MoVps13 with high confidence by AlphaFold2 software. Our results may be helpful for the development of specific targeted drugs to control fungal pathogens.

## 5. Conclusions

In this study, we identified the vacuolar protein-sorting receptor MoVps13 in *M. oryzae* and systematically analysed its biological function. We found that MoVps13 regulated hyphal growth, conidiation, and virulence. Expression of MoVps13 significantly decreased rates of fungal infection. In addition, we analysed the molecular mechanism of MoVps13 in autophagy, cell wall integrity, and sphingolipid synthesis. Our results uncovered the functions of MoVps13 in cell wall signalling, PM homeostasis, and ER-phagy in *M. oryzae*. Furthermore, we identified the SHR, Vps13_C and ATG_C domain structure of MoVps13 with high accuracy using alphaflod2. These results will facilitate better characterization of the pathogenetic mechanisms of rice blast fungus. Next, the precise molecular functions of MoVps13 protein should be investigated, which may serve as an experimental platform for drug testing based on protein structure. Future research will entail screening of specific anti-fungal compounds based on the 3D structure of MoVps13.

## Supplementary Materials

The following are available online at https://www.mdpi.com/article/10.3390/jof7121084/s1, Figure S1: The knockout strategies of MoVps13, Table S1: Primers used in this study.

## References

[1] Wilson R.A. (2021). Magnaporthe oryzae. Trends Microbiol..

[2] Cao H., Huang P., Yan Y., Shi Y., Dong B., Liu X., Ye L., Lin F., Lu J. (2018). The basic helix-loop-helix transcription factor Crf1 is required for development and pathogenicity of the rice blast fungus by regulating carbohydrate and lipid metabolism. Environ. Microbiol..

[3] Di Pietro A., Talbot N.J. (2017). Fungal pathogenesis: Combatting the oxidative burst. Nat. Microbiol..

[4] Hamer J.E., Howard R.J., Chumley F.G., Valent B. (1988). A mechanism for surface attachment in spores of a plant pathogenic fungus. Science.

[5] Fernandez J., Orth K. (2018). Rise of a Cereal Killer: The Biology of Magnaporthe oryzae Biotrophic Growth. Trends Microbiol..

[6] Rocha R.O., Elowsky C., Pham N.T.T., Wilson R.A. (2020). Spermine-mediated tight sealing of the Magnaporthe oryzae appressorial pore–rice leaf surface interface. Nat. Microbiol..

[7] Choi J., Kim Y., Kim S., Park J., Lee Y.-H. (2009). MoCRZ1, a gene encoding a calcineurin-responsive transcription factor, regulates fungal growth and pathogenicity of Magnaporthe oryzae. Fungal Genet. Biol..

[8] Ryder L.S., Talbot N.J. (2015). Regulation of appressorium development in pathogenic fungi. Curr. Opin. Plant. Biol..

[9] Foster A.J., Ryder L.S., Kershaw M.J., Talbot N.J. (2017). The role of glycerol in the pathogenic lifestyle of the rice blast fungus Magnaporthe oryzae. Environ. Microbiol..

[10] Talbot N.J. (2019). Appressoria. Curr. Biol..

[11] Zhu X.-M., Liang S., Shi H.-B., Lu J.-P., Dong B., Liao Q.-S., Lin F.-C., Liu X.-H. (2018). VPS9 domain-containing proteins are essential for autophagy and endocytosis in Pyricularia oryzae. Environ. Microbiol..

[12] Chen Y., Zhai S., Zhang H., Zuo R., Wang J., Guo M., Zheng X., Wang P., Zhang Z. (2014). Shared and distinct functions of two Gti1/Pac2 family proteins in growth, morphogenesis and pathogenicity ofMagnaporthe oryzae. Environ. Microbiol..

[13] Feng W., Yin Z., Wu H., Liu P., Liu X., Liu M., Yu R., Gao C., Zhang H., Zheng X. (2021). Balancing of the mitotic exit network and cell wall integrity signaling governs the development and pathogenicity in Magnaporthe oryzae. PLoS Pathog..

[14] Nie H.-Z., Zhang L., Zhuang H.-Q., Shi W.-J., Yang X.-F., Qiu D.-W., Zeng H.-M. (2019). The Secreted Protein MoHrip1 Is Necessary for the Virulence of Magnaporthe oryzae. Int. J. Mol. Sci..

[15] Giraldo M.C., Dagdas Y.F., Gupta Y.K., Mentlak T.A., Yi M., Martinez-Rocha A.L., Saitoh H., Terauchi R., Talbot N.J., Valent B. (2013). Two distinct secretion systems facilitate tissue invasion by the rice blast fungus Magnaporthe oryzae. Nat. Commun..

[16] Gao M., Yang H. (2018). VPS13: A lipid transfer protein making contacts at multiple cellular locations. J. Cell Biol..

[17] Rzepnikowska W., Flis K., Muñoz-Braceras S., Menezes R., Escalante R., Zoladek T. (2017). Yeast and other lower eukaryotic organisms for studies of Vps13 proteins in health and disease. Traffic.

[18] Bean B.D.M., Dziurdzik S.K., Kolehmainen K.L., Fowler C.M.S., Kwong W.K., Grad L.I., Davey M., Schluter C., Conibear E. (2018). Competitive organelle-specific adaptors recruit Vps13 to membrane contact sites. J. Cell Biol..

[19] Velayos-Baeza A., Vettori A., Copley R.R., Dobson-Stone C., Monaco A.P. (2004). Analysis of the human VPS13 gene family. Genomics.

[20] Hook S.C., Chadt A., Heesom K.J., Kishida S., Al-Hasani H., Tavare J.M., Thomas E.C. (2020). TBC1D1 interacting proteins, VPS13A and VPS13C, regulate GLUT4 homeostasis in C2C12 myotubes. Sci. Rep..

[21] Kumar N., Leonzino M., Hancock-Cerutti W., Horenkamp F.A., Li P., Lees J.A., Wheeler H., Reinisch K.M., De Camilli P. (2018). VPS13A and VPS13C are lipid transport proteins differentially localized at ER contact sites. J. Cell Biol..

[22] Mizuno E., Nakamura M., Agemura A., Kusumoto A., Ichiba M., Kurano Y., Muroya S., Sano A. (2007). Brain-specific transcript variants of 5′ and 3′ ends of mouse VPS13A and VPS13C. Biochem. Biophys. Res. Commun..

[23] Walker R.H. (2015). Untangling the Thorns: Advances in the Neuroacanthocytosis Syndromes. J. Mov. Disord..

[24] Douzgou S., Petersen M.B. (2011). Clinical variability of genetic isolates of Cohen syndrome. Clin. Genet..

[25] Momtazmanesh S., Rayzan E., Shahkarami S., Rohlfs M., Klein C., Rezaei N. (2020). A novel VPS13B mutation in Cohen syndrome: A case report and review of literature. BMC Med. Genet..

[26] Rodrigues J.M., Fernandes H.D., Caruthers C., Braddock S.R., Knutsen A.P. (2018). Cohen Syndrome: Review of the Literature. Cureus.

[27] Windholz J., Kovacs P., Tonjes A., Dittrich K., Bluher S., Kiess W., Stumvoll M., Korner A. (2011). Effects of genetic variants in ADCY5, GIPR, GCKR and VPS13C on early impairment of glucose and insulin metabolism in children. PLoS ONE.

[28] Nakada T.A., Boyd J.H., Russell J.A., Aguirre-Hernandez R., Wilkinson M.D., Thair S.A., Nakada E., McConechy M.K., Fjell C.D., Walley K.R. (2015). VPS13D Gene Variant Is Associated with Altered IL-6 Production and Mortality in Septic Shock. J. Innate Immun..

[29] John Peter A.T., Herrmann B., Antunes D., Rapaport D., Dimmer K.S., Kornmann B. (2017). Vps13-Mcp1 interact at vacuole–mitochondria interfaces and bypass ER–mitochondria contact sites. J. Cell Biol..

[30] Chen S., Mari M., Parashar S., Liu D., Cui Y., Reggiori F., Novick P.J., Ferro-Novick S. (2020). Vps13 is required for the packaging of the ER into autophagosomes during ER-phagy. Proc. Natl. Acad. Sci. USA.

[31] Bouatta N., Sorger P., Alquraishi M. (2021). Protein structure prediction by AlphaFold2: Are attention and symmetries all you need?. Acta Crystallogr. Sect. D Struct. Biol..

[32] Cramer P. (2021). AlphaFold2 and the future of structural biology. Nat. Struct. Mol. Biol..

[33] Su J., Zhang T., Wang P., Liu F., Tai G., Zhou Y. (2015). The water network in galectin-3 ligand binding site guides inhibitor design. Acta Biochim. Biophys. Sin..

[34] Jumper J., Evans R., Pritzel A., Green T., Figurnov M., Ronneberger O., Tunyasuvunakool K., Bates R., Zidek A., Potapenko A. (2021). Highly accurate protein structure prediction with AlphaFold. Nature.

[35] Zhu S., Yan Y., Qu Y., Wang J., Feng X., Liu X., Lin F., Lu J. (2021). Role refinement of melanin synthesis genes by gene knockout reveals their functional diversity in Pyricularia oryzae strains. Microbiol. Res..

[36] Talbot N.J., Ebbole D.J., Hamer J.E. (1993). Identification and characterization of MPG1, a gene involved in pathogenicity from the rice blast fungus Magnaporthe grisea. Plant Cell.

[37] Wei Y.-Y., Liang S., Zhang Y.-R., Lu J.-P., Lin F.-C., Liu X.-H. (2020). MoSec61β, the beta subunit of Sec61, is involved in fungal development and pathogenicity, plant immunity, and ER-phagy in Magnaporthe oryzae. Virulence.

[38] Lu J., Cao H., Zhang L., Huang P., Lin F. (2014). Systematic Analysis of Zn2Cys6 Transcription Factors Required for Development and Pathogenicity by High-Throughput Gene Knockout in the Rice Blast Fungus. PLoS Pathog..

[39] Muñoz-Braceras S., Calvo R., Escalante R. (2015). TipC and the chorea-acanthocytosis protein VPS13A regulate autophagy inDictyosteliumand human HeLa cells. Autophagy.

[40] Funato K., Riezman H., Muniz M. (2020). Vesicular and non-vesicular lipid export from the ER to the secretory pathway. Biochim. Biophys. Acta Mol. Cell Biol. Lipids.

[41] Qu Y., Wang J., Zhu X., Dong B., Liu X., Lu J., Lin F. (2020). The P5-type ATPase Spf1 is required for development and virulence of the rice blast fungus Pyricularia oryzae. Curr. Genet..

[42] De M., Oleskie A.N., Ayyash M., Dutta S., Mancour L., Abazeed M.E., Brace E.J., Skiniotis G., Fuller R.S. (2017). The Vps13p–Cdc31p complex is directly required for TGN late endosome transport and TGN homotypic fusion. J. Cell Biol..

[43] Zhu X.-M., Li L., Cai Y.-Y., Wu X.-Y., Shi H.-B., Liang S., Qu Y.-M., Naqvi N.I., Del Poeta M., Dong B. (2020). A VASt-domain protein regulates autophagy, membrane tension, and sterol homeostasis in rice blast fungus. Autophagy.

[44] Zhu X.-M., Li L., Wu M., Liang S., Shi H.-B., Liu X.-H., Lin F.-C. (2019). Current opinions on autophagy in pathogenicity of fungi. Virulence.

[45] Klionsky D.J., Abdel-Aziz A.K., Abdelfatah S., Abdellatif M., Abdoli A., Abel S., Abeliovich H., Abildgaard M.H., Abudu Y.P., Acevedo-Arozena A. (2021). Guidelines for the use and interpretation of assays for monitoring autophagy. Autophagy.

[46] Li L., Zhu X.-M., Su Z.-Z., Del Poeta M., Liu X.-H., Lin F.-C. (2021). Insights of roles played by septins in pathogenic fungi. Virulence.

[47] Nadal M., Gold S.E. (2010). The autophagy genes ATG8 and ATG1 affect morphogenesis and pathogenicity in Ustilago maydis. Mol. Plant Pathol..

[48] Lv W., Wang C., Yang N., Que Y., Talbot N.J., Wang Z. (2017). Genome-wide functional analysis reveals that autophagy is necessary for growth, sporulation, deoxynivalenol production and virulence in Fusarium graminearum. Sci. Rep..

[49] Wilkinson S. (2020). Emerging Principles of Selective ER Autophagy. J. Mol. Biol..

[50] Zaffagnini G., Martens S. (2016). Mechanisms of Selective Autophagy. J. Mol. Biol..

[51] Park J.-S., Neiman A.M. (2012). VPS13 Regulates Membrane Morphogenesis During Sporulation in Saccharomyces cerevisiae. J. Cell Sci..

[52] Sun L.X., Qian H., Liu M.Y., Wu M.H., Wei Y.Y., Zhu X.M., Lu J.P., Lin F.C., Liu X.H. (2021). Endosomal sorting complexes required for transport-0 (ESCRT-0) are essential for fungal development, pathogenicity, autophagy and ER-phagy in Magnaporthe oryzae. Environ. Microbiol..

[53] Liu X.-H., Chen S.-M., Gao H.-M., Ning G.-A., Shi H.-B., Wang Y., Dong B., Qi Y.-Y., Zhang D.-M., Lu G.-D. (2015). The small GTPase MoYpt7 is required for membrane fusion in autophagy and pathogenicity ofMagnaporthe oryzae. Environ. Microbiol..

[54] Sakulkoo W., Osés-Ruiz M., Oliveira Garcia E., Soanes D.M., Littlejohn G.R., Hacker C., Correia A., Valent B., Talbot N.J. (2018). A single fungal MAP kinase controls plant cell-to-cell invasion by the rice blast fungus. Science.

[55] Zhang X., Bian Z., Xu J.-R. (2018). Assays for MAP Kinase Activation in Magnaporthe oryzae and Other Plant Pathogenic Fungi. Plant Pathogenic Fungi and Oomycetes.

[56] Jiang C., Zhang X., Liu H., Xu J.-R. (2018). Mitogen-activated protein kinase signaling in plant pathogenic fungi. PLoS Pathog..

[57] Xu J.R., Staiger C.J., Hamer J.E. (1998). Inactivation of the mitogen-activated protein kinase Mps1 from the rice blast fungus prevents penetration of host cells but allows activation of plant defense responses. Proc. Natl. Acad. Sci. USA.

[58] Zhou T., Dagdas Y.F., Zhu X., Zheng S., Chen L., Cartwright Z., Talbot N.J., Wang Z. (2017). The glycogen synthase kinase MoGsk1, regulated by Mps1 MAP kinase, is required for fungal development and pathogenicity in Magnaporthe oryzae. Sci. Rep..

[59] Sun G., Elowsky C., Li G., Wilson R.A. (2018). TOR-autophagy branch signaling via Imp1 dictates plant-microbe biotrophic interface longevity. PLoS Genet..

[60] Qian B., Liu X., Ye Z., Zhou Q., Liu P., Yin Z., Wang W., Zheng X., Zhang H., Zhang Z. (2021). Phosphatase-associated protein MoTip41 interacts with the phosphatase MoPpe1 to mediate crosstalk between TOR and cell wall integrity signalling during infection by the rice blast fun. Environ. Microbiol..

[61] Paluch E., Heisenberg C.P. (2009). Biology and physics of cell shape changes in development. Curr. Biol..

[62] Tafesse F.G., Holthuis J.C. (2010). Cell biology: A brake on lipid synthesis. Nature.

[63] Gonzalez-Solis A., Han G., Gan L., Li Y., Markham J.E., Cahoon R.E., Dunn T.M., Cahoon E.B. (2020). Unregulated Sphingolipid Biosynthesis in Gene-Edited Arabidopsis ORM Mutants Results in Nonviable Seeds with Strongly Reduced Oil Content. Plant Cell.

[64] Guan X.L., Souza C.M., Pichler H., Dewhurst G., Schaad O., Kajiwara K., Wakabayashi H., Ivanova T., Castillon G.A., Piccolis M. (2009). Functional Interactions between Sphingolipids and Sterols in Biological Membranes Regulating Cell Physiology. Mol. Biol. Cell.

[65] Hurst L.R., Fratti R.A. (2020). Lipid Rafts, Sphingolipids, and Ergosterol in Yeast Vacuole Fusion and Maturation. Front. Cell Dev. Biol..

[66] Yang H., Tong J., Lee C.W., Ha S., Eom S.H., Im Y.J. (2015). Structural mechanism of ergosterol regulation by fungal sterol transcription factor Upc2. Nat. Commun..

[67] He M., Su J., Xu Y., Chen J., Chern M., Lei M., Qi T., Wang Z., Ryder L.S., Tang B. (2020). Discovery of broad-spectrum fungicides that block septin-dependent infection processes of pathogenic fungi. Nat. Microbiol..

[68] Fisher M.C., Hawkins N.J., Sanglard D., Gurr S.J. (2018). Worldwide emergence of resistance to antifungal drugs challenges human health and food security. Science.

