# Supplementary material for: Vacuolar Protein-Sorting Receptor MoVps13 Regulates Conidiation and Pathogenicity in Rice Blast Fungus Magnaporthe oryzae
Source: J Fungi (Basel). 2021 Dec 17;7(12):1084. doi: 10.3390/jof7121084 (PMC8708568; doi:10.3390/jof7121084)
Supplement: Supplementary file 1 [file jof-07-01084-s001.zip › jof-1490378-supplementary.pdf]

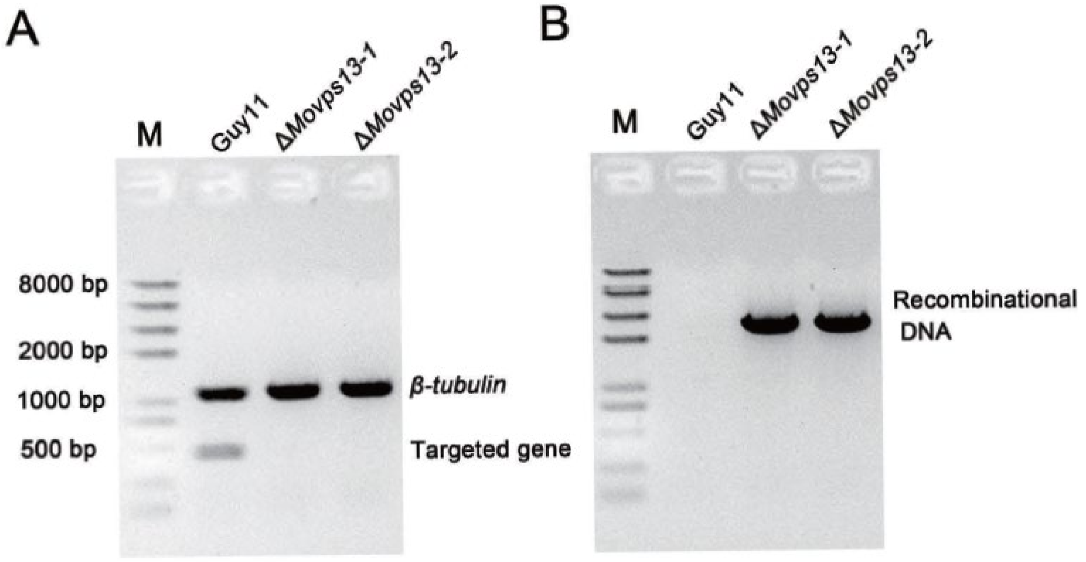

Figure S1. The knockout strategies of MoVps13. (**A**) The transformants were screened by PCR for the targeted gene using the β-tubulin gene as a positive control. A characteristic band was amplified from the wild-type strain, indicating the presence of targeted gene, whereas this band was absent from the null mutants. (**B**) The transformants were screened by PCR for a unique recombinational DNA fragment marked as a knockout event. A 1.2-2.0 kb band was amplified from the null mutants, whereas this band was absent from the wild-type strain.

Table S1 Primers used in this study

Gene Name	Sequences
MoVps13_upF:	AGGCTAACTGACACTCTAGAACAATGGGCTCGAAGAACTGGT
MoVps13_upR:	TGTTGACCTCCACTACTTAGAAGTCAAGGCAGC
MoVps13_downF:	GGAATAGAGTAGATGGTTTAGCCCTGTGGACTGTTCT
MoVps13_downR:	CGACGGCCAGTGCCAAGCTTCTGACCCAAGCTCAACATGCC
MoVps13_upyzF:	GTGCCTTCTCTGCATATT
MoVps13_innerF:	GATCCTGCCCAGCTCAAAGTC
MoVps13_innerR:	GCTGACGGCGCTGAATTCCTTG
MoVps13_Pkd5_GFPF:	ATCAATCACAATGGCCATGTTGGAAGGGCTTGTGGC
MoVps13_Pkd5_GFPR:	CGCCCTTGCTCACCATCTCCAACGCTCTGAAGCGCCGAT
Tubulin_F	CCCATCGAAGCCCTTCGAAGG
Tubulin_R	CTCAACGACGGTGTCGGAAACC
Sec63_Pkd5_GFPF:	ATCAATCACAATGGCCATGAGTAGCGACTACTCATACG
Sec63_Pkd5_GFPR:	CGCCCTTGCTCACCATAGACTCATCCTCAGTGTC
